# Epigenetic alterations in creatine transporter deficiency: a new marker for dodecyl creatine ester therapeutic efficacy monitoring

**DOI:** 10.3389/fnins.2024.1362497

**Published:** 2024-04-17

**Authors:** Léa Broca-Brisson, Clémence Disdier, Rania Harati, Rifat Hamoudi, Aloïse Mabondzo

**Affiliations:** ^1^Département Médicaments et Technologies pour la Santé, CEA, INRAE, SPI, Université Paris-Saclay, Gif-sur-Yvette, France; ^2^Ceres Brain Therapeutics, ICM-Hôpital Pitié-Salpétrière, Paris, France; ^3^Department of Pharmacy Practice and Pharmacotherapeutics, College of Pharmacy, University of Sharjah, Sharjah, United Arab Emirates; ^4^Research Institute for Medical and Health Sciences, University of Sharjah, Sharjah, United Arab Emirates; ^5^Department of Clinical Sciences, College of Medicine, University of Sharjah, Sharjah, United Arab Emirates; ^6^Center of Excellence of Precision Medicine, Research Institute of Medical and Health Sciences, University of Sharjah, Sharjah, United Arab Emirtes; ^7^Division of Surgery and Interventional Science, University College London, London, United Kingdom; ^8^ASPIRE Precision Medicine Research Institute Abu Dhabi, University of Sharjah, Sharjah, United Arab Emirates; ^9^Center of Excellence of Precision Medicine, Research Institute of Medical and Health Sciences, University of Sharjah, Sharjah, United Arab Emirates; ^10^BIMAI-Lab, Biomedically Informed Artificial Intelligence Laboratory, University of Sharjah, Sharjah, United Arab Emirates

**Keywords:** creatine transporter deficiency, epigenetic, methylation, cerebral organoids, dodecyl creatine ester

## Abstract

Creatine transporter deficiency (CTD) is an X-linked disease caused by mutations in the *Slc6a8* gene. The impaired creatine uptake in the brain leads to developmental delays with intellectual disability. We hypothesized that deficient creatine uptake in CTD cerebral cells impact methylation balance leading to alterations of genes and proteins expression by epigenetic mechanism. In this study, we determined the status of nucleic acid methylation in both *Slc6a8* knockout mouse model and brain organoids derived from CTD patients’ cells. We also investigated the effect of dodecyl creatine ester (DCE), a promising prodrug that increases brain creatine content in the mouse model of CTD. The level of nucleic acid methylation was significantly reduced compared to healthy controls in both *in vivo* and *in vitro* CTD models. This hypo-methylation tended to be regulated by DCE treatment *in vivo*. These results suggest that increased brain creatine after DCE treatment restores normal levels of DNA methylation, unveiling the potential of using DNA methylation as a marker to monitor the drug efficacy.

## 1 Introduction

Creatine transporter deficiency (CTD) is an inborn error of creatine metabolism in which creatine (Cr) is not properly transported to the brain and muscles due to defective creatine transporters (CrT). Patients with CTD express speech and behavior abnormalities, intellectual disabilities, development delay, seizures, and autistic behavior due to lack of brain Cr as energy buffer (Braissant et al., 2011). There is no pharmacological treatment to alleviate behavioral symptoms of CTD (Fernandes-Pires and Braissant, 2022). There is an urgent need to develop new therapeutic strategies and new relevant tools such as translational models and markers to evaluate therapeutic efficacy and speed up drug development.

Cr plays a central role in brain energetics (Fernandes-Pires and Braissant, 2022). Cerebral Cr originates from diet after transport across the blood brain barrier and cerebral cell membranes or from *de novo* synthesis. In CTD, the peripheral Cr is not distributed to the brain cells due to the non-functional CrT. Consequently, the Cr store of CTD patient brain cells only rely on *de novo* cerebral synthesis. CTD brain cells may try to increase their Cr synthesis to compensate the energy unbalance even if cerebral cells rarely express both enzymes (AGAT and GAMT) needed for Cr *de novo* synthesis (Braissant and Henry, 2008; Braissant et al., 2010). Indeed, this upregulation of Cr biosynthesis was previously demonstrated in the muscle of CrT knockout (KO) mice (Russell et al., 2014; Stockebrand et al., 2018). Cr *de novo* synthesis need S-adenosylmethionine (SAM) as a methyl donor, this group being transfer to guanidinoacetate by guanidinoacetate N-methyltransferase (GAMT) in the second step (Curt et al., 2015). This last step consume approximately 40% of methyl groups supplied by SAM and consequently places a significant burden on the labile methyl groups store (Stead et al., 2001; Brosnan et al., 2011). In this study, we hypothesized that the methyl unbalance could lead to various consequences including altered DNA and RNA methylation since DNA and RNA methyltransferase also use SAM as precursor.

Organoids are becoming increasingly important in the field of neurobiology and drug discovery, since they allow enhancement of modelling of human tissues and they can provide greater insight into the mechanisms of human development and disease. We recently implement and characterized cerebral organoids from CTD patient cells (Broca-Brisson et al., 2023). This innovative model open opportunities for physiopathology mechanistic studies and for drug discovery.

In order to validate the hypothesis of methylation unbalance we looked at nucleic acid methylation status in both CrT knockout mouse model and brain organoids derived from CTD patients. In an intent to assess the use as possible marker of treatment efficacy, mice were also exposed to dodecyl creatine ester (DCE). DCE, as a Cr prodrug, was demonstrated as a promising drug to treat CTD after administration by the nasal route taking advantage of the nose-to-brain pathway (Ullio-Gamboa et al., 2019; Mabondzo et al., 2023).

## 2 Materials and methods

### 2.1 Animals and intranasal treatment

All procedures were in accordance with European directives on the protection and use of laboratory animals (Council Directive 2010/63/UE, French decree 2013-118). The experimental protocol was evaluated and validated by a local ethic committee for animal use and approved by the French government (n° APAFIS#7466-2016110417049220).

Male 12–16 weeks old CrT^–/*y*^ and CrT^+/*y*^ mice were generated on the C57BL/6J background as previously described (Skelton et al., 2011). Littermate WTs were used as controls. The mice were housed at 22°C on a 12–12 h light–dark cycle and provided food and water *ad libitum*.

Dodecyl creatine ester (DCE) was synthesized as described previously (Trotier-Faurion et al., 2013). The emulsion formulation comprised 22.3% w/w of Montanox 80 (SEPPIC, Paris, France), 19% w/w of kollisolv MCT70 (BASF, Ludwigshafen, Germany) and 56.7% w/w of regular saline. The amount of DCE (2% w/w) was first suspended in the mix of oil and surfactant with magnetic stirring at room temperature. The premix and the saline were preheat at 40°C. Finally, a fixed amount of preheat regular saline was added to the above mixture and stirred continuously for 10 min until a homogenous emulsion was obtained. The emulsion was kept under stirring during the cooling phase then store at 4°C until use. The vehicle was prepare with the same process with replacement of the 2% w/w of DCE by regular saline.

Dodecyl creatine ester was intra-nasally administered to CrT KO mice for 30 days, while wild-type (WT) and vehicle-treated CrT KO mice were used as controls. Using a P20 micropipette, 6 μl of DCE formulation or vehicle was placed in the nostril. The intranasal treatment DCE (2% w/w) or vehicle was given twice bilaterally (24 μl total volume) for 30 days. In the DCE treated group, each mouse received 0.48 mg of DCE daily.

### 2.2 iPSC culture and Brain organoids generation

Healthy BJ and SP iPSC were a generous gift of Frank Yates (Pavoni et al., 2018). CTD iPSCs were obtained by reprogramming CTD patient’s fibroblasts in a previous study (Broca-Brisson et al., 2023). All the mutations and clinical features were previously described by Valayannopoulos et al. (2013).

All iPSC lines were maintained on hESC-qualified Matrigel (Corning, NY, USA) in mTeSR™1 medium (STEMCELL Technologies) and passaged using ReLeSR™ (STEMCELL Technologies, Vancouver, BC, Canada).

Brain organoids were generated as previously described (Lancaster and Knoblich, 2014; Nassor et al., 2020) with slight modifications (Broca-Brisson et al., 2023). Brain organoids were used at 2 month-old.

### 2.3 DNA extraction and 5-methylcytosine (5mC) quantification

In order to check the DNA methylation, Total DNA was extracted from 20 to 25 mg of total brain tissue or each brain organoids using the NucleoSpin Tissue Mini kit for DNA (Macherey-Nagel, Duren, Germany, 740952.50), according to manufacturer’s instructions. The purity and concentration of DNA were measured using the NanoDrop ND-1000 spectrophotometer at 230, 260 and 280 nm (NanoDrop Technologies, Wiligmington, DE, USA).

Global 5mC DNA content in brain tissue or brain organoids was quantified using the ELISA-based Methylated DNA Quantification Kit (Colorimetric) (Abcam ab117128, Cambridge, UK). The input DNA was diluted in TE buffer (Tris-EDTA buffer) to an optimum 100 ng per reaction. The assay was performed in duplicates according to the manufacturer’s instructions. Plates were read at 450 nm on a BioTek Epoch Microplate Spectrophotometer (Agilent Technologies, Santa Clara, CA, USA). The negative control absorbance was subtracted to all measurements for background absorbance correction. Level of DNA methylation were expressed as relative to the control group mean.

### 2.4 RNA extraction and 6-methyladenosine (m6A) quantification

To check the methylation at the RNA level, total RNA was isolated from brain organoids with RNeasy Plus Universal Tissue Mini kit (Agilent Technologies, Hilden, Germany) and Precellys Evolution tissue homogenizer (Bertin-Technology, Montigny Le Bretonneux, France). The concentration and purity of RNA samples were checked using the NanoDrop ND-1000 spectrophotometer at 260 and 280 nm (NanoDrop Technologies, Wiligmington, DE, USA); the A260/280 ratio ranged from 1.8 to 2.2.

Global m6A RNA content in brain organoids was quantified using the ELISA-based EpiQuik m6A RNA Methylation Quantification Kit (Colorimetric) (Epigentek P-9005, Farmingdale, NY, USA). The input RNA was diluted in TE buffer (Tris-EDTA buffer) to an optimum 200 ng per reaction. The assay was performed in duplicates according to the manufacturer’s instructions. Plate was read at 450 nm on a BioTek Epoch Microplate Spectrophotometer (Agilent Technologies, Santa Clara, CA, USA). The negative control absorbance was subtracted to all measurements for background absorbance correction. Level of RNA methylation were expressed as relative to the control group mean.

### 2.5 Statistics

Statistical analysis was performed using the GraphPad Prism 10.1 program. Experimental comparisons with multiple groups were analyzed using Kruskal–Wallis test with Dunn’s multiple comparisons test for *post hoc* analysis.

## 3 Results

To investigate our hypothesis of deficient nucleic acid methylation in CTD, we first used CrT KO mice, a model that recapitulates cognitive impairments and major pathophysiological aspects of CTD (Skelton et al., 2011; Udobi et al., 2018, 2019).

**FIGURE 1:**
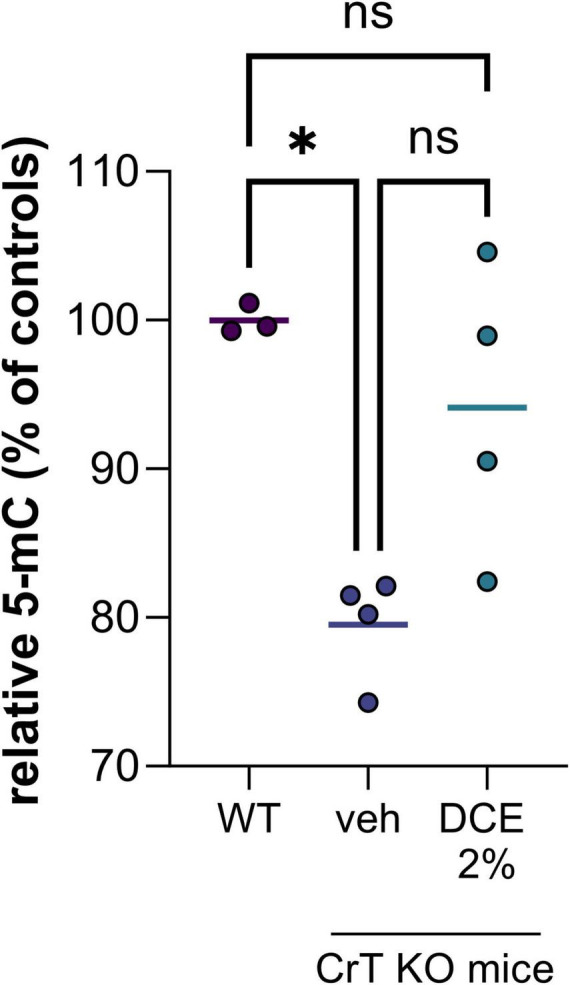
DNA methylation level in brain tissue of wild type (WT) and CrT KO mice with and without DCE treatment. *N* = 3–4 mice per group (WT = wild type; veh = CrT KO mice treated with the vehicle; DCE 2% = CrT KO mice treated with the dodecyl creatine ester formulation 2% w/w, 0.48 mg/day intranasally for 30 days). Data express as relative quantification to controls and analyzed using Kruskal–Wallis test with Dunn’s multiple comparisons test. *P ≤ 0.05.

In the nucleus, the most abundant modification of DNA is methylation on the 5th carbon of cytosines (5mC), mainly in the context of CpG dinucleotides. The presence of methylated cytosine in the promoter sequence of a gene is generally associated with transcriptional repression because it directly inhibits the binding of certain transcription factors and can also recruit repressors from the MBP (methyl binding proteins) family. We demonstrated a hypomethylation of DNA in CrT KO mice brain corresponding to a decrease of 5-methylcytosine (5mC) of about 20% compared to wild type mice (Figure 1). This hypomethylation of DNA could impact gene expression leading to cognitive dysfunctions observed in the CrT KO mice model.

Dodecyl creatine ester administered by the nasal route is a promising therapeutic option to replenish creatine level in CTD patient brain cells. We previously demonstrated the efficacy of this strategy *in vivo* by showing improvement of cognitive functions, restoration of the expression of keys molecular markers and increase cerebral Cr in well-characterized CTD mouse models (Ullio-Gamboa et al., 2019; Mabondzo et al., 2023). In this study, we evaluate the potential of DNA hypomethylation as a biomarker of therapeutic efficacy. Our findings are confounded by the relatively low number of mice in each group as well as the heterogeneity among mice. Despite the high heterogeneity among CrT KO mice, the results showed that 0.48 mg of intranasal DCE daily for 30 days tends to restore DNA methylation level in the brain of CrT KO mice (Figure 1). This observation demonstrated that restauration of Cr in the brain can impact DNA methylation and potentially a wide variety of gene expression.

**FIGURE 2:**
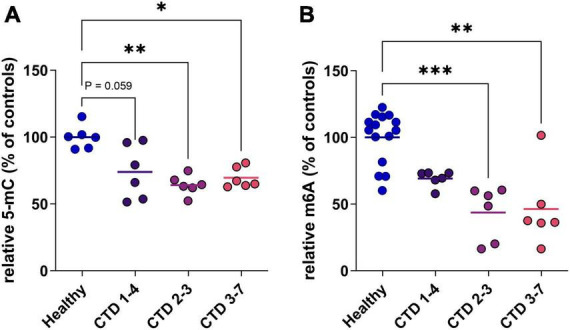
DNA and RNA methylation level in CTD brain organoids (CTD 1–4, 2–3 and 3–7) vs. healthy brain organoids. **(A)** 5-mC in DNA (*n* = 6). **(B)** m6A in mRNA (*n* = 6–14). Data express as relative quantification to controls, and analyzed using Kruskal–Wallis test with Dunn’s multiple comparisons test. *P < 0.05; **P < 0.01; ***P < 0.001.

In a second step, to evaluate the potential translation of this marker in a human model, we used a CTD brain organoid model characterized previously (Broca-Brisson et al., 2023). We highlight a reduction of 5mC in DNA of all CTD brain organoids from 3 patients (CTD 1–4, 2–3 and 3–7) compared to healthy organoids (Figure 2A). This hypomethylation corresponds to a 31, 43 and 36% decrease of 5mC in DNA in CTD 1–4, 2–3 and 3–7, respectively (Figure 2A).

RNA can be the target of various modifications of its constituent nucleotides, allowing very fine control of protein synthesis. In mammals, the most frequent modification is the addition of a methyl group at position 6 of adenosine residues (m6A). We complete analysis of methylation status in CTD brain organoids by investigating RNA methylation. The profile of RNA methylation is similar to results obtained on DNA, with a decrease of m6A levels in CTD brain organoids corresponding to about 30 to 54% decrease depending on patients (Figure 2B).

## 4 Discussion

Patho-physiological mechanisms implicated in CTD remain understudy while Cr play a pivotal role in energy metabolism and is implicated in many cellular processes, especially in the brain (Fernandes-Pires and Braissant, 2022). In this study, we used both CrT KO mice and CTD human brain organoids to investigate the epigenetic changes in CTD pathology. In addition, we evaluate how DCE, a promising Cr prodrug can ameliorate this marker *in vivo*.

**FIGURE 3:**
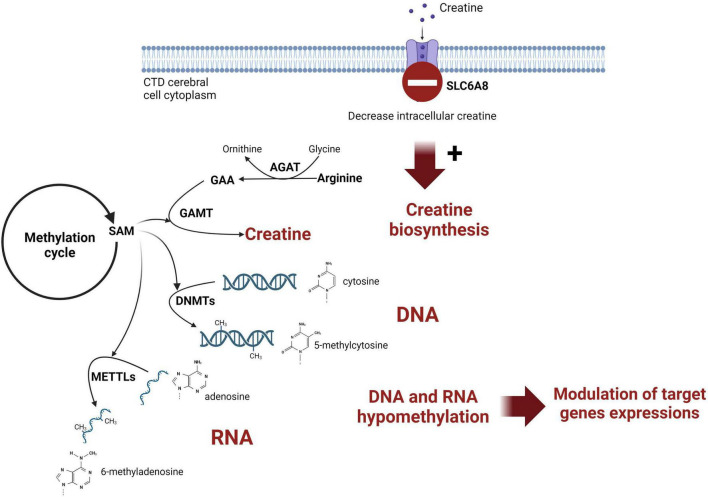
Schematic representation of the link between creatine intracerebral synthesis and DNA methylation in CTD patient brain. SAM, S-adenosylmethionine; AGAT, glycine amidinotransferase; GAMT, guanidinoacetate methyltransferase; DNMT, DNA methyltransferase; METTL, methyltransferase-like; GAA, guanidinoacetic acid.

Creatine (Cr) *de novo* synthesis places a huge burden on methyl balance (Stead et al., 2001; Brosnan et al., 2011). The implication of Cr synthesis on the methylation cycle and main findings are schematized on Figure 3. Methylation plays a pivotal role in the regulation of biological processes since it is intricately involved in modifying nucleic acids (DNA and RNA) and histones. The privileged site for DNA methylation is at the C5 position of cytosine (5mC) within CpG dinucleotides (CpG sites). For RNA, m6A (N6-methyladenosine) is the most abundant RNA modification.

In this study, we highlighted a 5mC and m6A decrease in CTD cerebral cells using both CrT KO mice model and CTD patient derived brain organoids. Methylation of nucleic acids is one of several epigenetic mechanisms that cells can use to control gene expression which play key regulatory roles in organogenesis, homeostasis and pathological processes (Yao et al., 2016). In the central nervous system, DNA methylation is crucial for normal brain development and functioning (Younesian et al., 2022). Recent studies have pointed to the role of epigenetic changes such as DNA, RNA methylation or histone modifications in various neurological disorders (Younesian et al., 2022). As example, observations in patients suffering of autism spectrum disorders, that share similar symptomatology with CTD, highlight the importance of DNA methylation in this disease (Williams and LaSalle, 2022). The DNA and RNA hypomethylation observed in both CrT KO mice and CTD brain organoids validates our hypothesis of methylation dysregulation and could be related to the CTD symptomatology. Three patients cell lines were used to generate CTD patient derived brain organoids. The level of hypomethylation observed *in vitro* is the reflection of the heterogeneity of CTD symptomatology in humans. Thus, the model employed in this study provides the possibility of studying this heterogeneity in a more translatable model.

Our hypothesis was that endogenous synthesis of Cr is stimulated in CTD cerebral cells in an attempt to restore creatine pool. The adaptative response may impact SAM abundance. It will be interesting to evaluate SAM levels and the other metabolites of the methylation cycle (e.g., homocysteine and S-adenosylhomocysteine) to confirm this mechanism. Investigation of the expression and activity of the enzymes implicated in methylation processes may also complete the description of the impact of the lack of Cr. In addition, it will be interesting to look at other epigenetic processes such as methylation and acetylation of histones.

We established previously that DCE, a prodrug of Cr, improves Cr cerebral levels and cognitive functions of CrT KO mice after one month of intranasal administration (Ullio-Gamboa et al., 2019; Mabondzo et al., 2023). Despite the variability in the DCE treated group and the small number of animals included in the analysis, improvement of the methylation after treatment with DCE in CrT KO suggests the use of nucleic acid methylation as a potential biomarker of treatment efficacy and could complement the potential efficacy in the biomarkers previously highlighted (Mabondzo et al., 2023).

Adult mice were used in this study. However, since epigenetic mechanisms such as nucleic acids methylation have a key role pathological process and CTD symptomatology appears in the early childhood, it will be interesting to design experiments in younger animals.

In addition, this DNA hypomethylation is a potential target for adjunctive therapies. Indeed, the SAM supply relies on the one-carbon cycle in which enter folate, vitamin B12, vitamin B2, and other precursors such as serine, glycine, histidine, tryptophan and choline. Even after restoring Cr cerebral levels, CTD patients may benefit from dietary supplementation with known methyl donors such as betaine, methionine or precursors of SAM that will balance the available methyl donor levels.

Today, several CrT knockout rodent models are available and recapitulate many aspects of the human pathophysiology including cognitive disorders (Skelton et al., 2011; Baroncelli et al., 2014; Duran-Trio et al., 2022). Those models are used for pre-clinical investigations but development of new therapeutic strategy is slow down by the lack of easily quantifiable markers to monitor pharmacological efficacy. In addition to rodent models, human cell-based organoids are recently allow to study human-specific aspects of the physiopathology and may offer new opportunities to better understand molecular pathways involved in the disease, discover new markers, and potential targets (Broca-Brisson et al., 2023). Consistency of the observations in *in vivo* model and CTD patient brain organoids confirm the translational potential of this *in vitro* model for preclinical pharmacology studies.

The study has several limitations. The CTD brain organoids were generated from three families. As male patients’ mutations in this study are a deletion of one amino acid, it could be interesting to generate CTD brain organoids from cells with other types of mutations as well as brain organoids from female patients that have a milder clinical phenotype to evaluate the landscape epigenetic alteration on the CTD pathophysiology. In addition, future study is required to investigate the effect of DCE on the methylation status in CTD brain organoids. Such a study will need an optimization of the experimental protocol due to the instability of DCE in the cell culture medium of brain organoids.

Overall, using mouse model of CTD and human cell based brain organoids, we demonstrate for the first time the implication of epigenetic alterations in the pathophysiology of CTD. Increase Cr in the brain of CrT KO mice after DCE treatment tend to improve DNA and RNA methylation levels, which unveils the potential of using DNA methylation as a marker to monitor the drug efficacy.

## Data availability statement

The original contributions presented in the study are publicly available. This data can be found here: Dryad, https://datadryad.org/stash/share/NvDFLckFGBNfJhdZS4QLCrqoh9MuR5UqOmUa0j81sAo.

## Ethics statement

The studies involving humans were approved by the Advisory Committee for the Protection of Persons in Biomedical Research Cochin Hospital, Paris, n°18-05. Human fibroblasts from CTD patients, were obtained from skin biopsy specimens and were a gift from the Centre de Référence des Maladies Héréditaires du Métabolisme at the Necker Hospital in Paris. Three patients with cerebral creatine deficiency caused by lack of creatine transporter were studied. Informed and a written consent was obtained from all anonymized human CTD subjects, and experiments were carried out in accordance with relevant guidelines and regulations. All the mutations were previously described by Valayannopoulos et al. (2013). The studies were conducted in accordance with the local legislation and institutional requirements. The human samples used in this study were acquired from another research group. Written informed consent for participation was not required from the participants or the participants’ legal guardians/next of kin in accordance with the national legislation and institutional requirements. The animal study was approved by APAFIS #24482-2020030411173844 v3.

## Author contributions

LB-B: Formal analysis, Investigation, Methodology, Writing – original draft. CD: Data curation, Formal analysis, Investigation, Methodology, Validation, Writing – review & editing. RH: investigation, Witing – review & editing. RiH: Conceptualization, Funding acquisition, Validation, Writing – review & editing. AM: Conceptualization, Data curation, Funding acquisition, Investigation, Methodology, Project administration, Resources, Supervision, Validation, Writing – original draft, Writing – review & editing.

## References

[B1] Baroncelli L. Alessandri M. G. Tola J. Putignano E. Migliore M. Amendola E. (2014). A novel mouse model of creatine transporter deficiency. *F1000Res* 3:228. 10.12688/f1000research.5369.1 25485098 PMC4243761

[B2] Braissant O. Beard E. Torrent C. Henry H. (2010). Dissociation of AGAT, GAMT and SLC6A8 in CNS: Relevance to creatine deficiency syndromes. *Neurobiol. Dis.* 37 423–433. 10.1016/j.nbd.2009.10.022 19879361

[B3] Braissant O. Henry H. (2008). AGAT, GAMT and SLC6A8 distribution in the central nervous system, in relation to creatine deficiency syndromes: A review. *J. Inherit. Metab. Dis.* 31 230–239. 10.1007/s10545-008-0826-9 18392746

[B4] Braissant O. Henry H. Beard E. Uldry J. (2011). Creatine deficiency syndromes and the importance of creatine synthesis in the brain. *Amino Acids* 40 1315–1324. 10.1007/s00726-011-0852-z 21390529

[B5] Broca-Brisson L. Harati R. Disdier C. Mozner O. Gaston-Breton R. Maïza A. (2023). Deciphering neuronal deficit and protein profile changes in human brain organoids from patients with creatine transporter deficiency. *eLife* 12:R88459. 10.7554/elife.88459.1 PMC10575631 37830910

[B6] Brosnan J. T. da Silva R. P. Brosnan M. E. (2011). The metabolic burden of creatine synthesis. *Amino Acids* 40 1325–1331. 10.1007/s00726-011-0853-y 21387089

[B7] Curt M. J. C. Voicu P. M. Fontaine M. Dessein A. F. Porchet N. Mention-Mulliez K. (2015). Creatine biosynthesis and transport in health and disease. *Biochimie* 119 146–165. 10.1016/j.biochi.2015.10.022 26542286

[B8] Duran-Trio L. Fernandes-Pires G. Grosse J. Soro-Arnaiz I. Roux-Petronelli C. Binz P. A. (2022). Creatine transporter-deficient rat model shows motor dysfunction, cerebellar alterations, and muscle creatine deficiency without muscle atrophy. *J. Inherit. Metab. Dis.* 45 278–291. 10.1002/jimd.12470 34936099 PMC9302977

[B9] Fernandes-Pires G. Braissant O. (2022). Current and potential new treatment strategies for creatine deficiency syndromes. *Mol. Genet. Metab.* 135 15–26. 10.1016/j.ymgme.2021.12.005 34972654

[B10] Lancaster M. A. Knoblich J. A. (2014). Generation of cerebral organoids from human pluripotent stem cells. *Nat. Protoc.* 9 2329–2340. 10.1038/nprot.2014.158 25188634 PMC4160653

[B11] Mabondzo A. Harati R. Broca-Brisson L. Guyot A.-C. Costa N. Cacciante F. (2023). Dodecyl creatine ester improves cognitive function and identifies key protein drivers including KIF1A and PLCB1 in a mouse model of creatine transporter deficiency. *Front. Mol. Neurosci.* 16:1118707. 10.3389/fnmol.2023.1118707 37063368 PMC10103630

[B12] Nassor F. Jarray R. Biard D. S. F. Maïza A. Papy-Garcia D. Pavoni S. (2020). Long term gene expression in human induced pluripotent stem cells and cerebral organoids to model a neurodegenerative disease. *Front. Cell Neurosci.* 14:14. 10.3389/fncel.2020.00014 32116560 PMC7026130

[B13] Pavoni S. Jarray R. Nassor F. Guyot A. C. Cottin S. Rontard J. (2018). Small-molecule induction of A beta-42 peptide production in human cerebral organoids to model Alzheimer’s disease associated phenotypes. *PLoS One* 13:e0209150. 10.1371/journal.pone.0209150 30557391 PMC6296660

[B14] Russell A. P. Ghobrial L. Wright C. R. Lamon S. Brown E. L. Kon M. (2014). Creatine transporter (SLC6A8) knockout mice display an increased capacity for in vitro creatine biosynthesis in skeletal muscle. *Front. Physiol.* 5:314. 10.3389/fphys.2014.00314 25206338 PMC4144344

[B15] Skelton M. R. Schaefer T. L. Graham D. L. Degrauw T. J. Clark J. F. Williams M. T. (2011). Creatine transporter (CrT; Slc6a8) knockout mice as a model of human CrT deficiency. *PLoS One* 6:e16187. 10.1371/journal.pone.0016187 21249153 PMC3020968

[B16] Stead L. M. Au K. P. Jacobs R. L. Brosnan M. E. Brosnan J. T. (2001). Methylation demand and homocysteine metabolism: Effects of dietary provision of creatine and guanidinoacetate. *Am. J. Physiol. Endocrinol. Metab.* 281 E1095–E1100. 11595668 10.1152/ajpendo.2001.281.5.E1095

[B17] Stockebrand M. Sasani A. Das D. Hornig S. I Hermans-Borgmeyer H. A. Lake D. I. (2018). A mouse model of creatine transporter deficiency reveals impaired motor function and muscle energy metabolism. *Front. Physiol.* 9:773. 10.3389/fphys.2018.00773 30013483 PMC6036259

[B18] Trotier-Faurion A. Dezard S. Taran F. Valayannopoulos V. de Lonlay P. Mabondzo A. (2013). Synthesis and biological evaluation of new creatine fatty esters revealed dodecyl creatine ester as a promising drug candidate for the treatment of the creatine transporter deficiency. *J. Med. Chem.* 56 5173–5181. 10.1021/jm400545n 23697594

[B19] Udobi K. C. Delcimmuto N. Kokenge A. N. Abdulla Z. I. Perna M. K. Skelton M. R. (2019). Deletion of the creatine transporter gene in neonatal, but not adult, mice leads to cognitive deficits. *J. Inherit. Metab. Dis.* 42 966–974. 10.1002/jimd.12137 31209903 PMC6739135

[B20] Udobi K. C. Kokenge A. N. Hautman E. R. Ullio G. Coene J. Williams M. T. (2018). Cognitive deficits and increases in creatine precursors in a brain-specific knockout of the creatine transporter gene Slc6a8. *Genes Brain Behav.* 17:e12461. 10.1111/gbb.12461 29384270 PMC6696915

[B21] Ullio-Gamboa G. Udobi K. C. Dezard S. Perna M. K. Miles K. N. Costa N. (2019). Dodecyl creatine ester-loaded nanoemulsion as a promising therapy for creatine transporter deficiency. *Nanomedicine* 14 1579–1593. 10.2217/nnm-2019-0059 31038003 PMC6613044

[B22] Valayannopoulos V. Bakouh N. Mazzuca M. Nonnenmacher L. Hubert L. Makaci F. L. (2013). Functional and electrophysiological characterization of four non-truncating mutations responsible for creatine transporter (SLC6A8) deficiency syndrome. *J. Inherit. Metab. Dis.* 36 103–112. 10.1007/s10545-012-9495-9 22644605

[B23] Williams L. A. LaSalle J. M. (2022). Future prospects for epigenetics in autism spectrum disorder. *Mol. Diagn. Ther.* 26 569–579. 10.1007/s40291-022-00608-z 35962910 PMC9626414

[B24] Yao B. Christian K. M. He C. Jin P. Ming G. L. Song H. (2016). Epigenetic mechanisms in neurogenesis. *Nat. Rev. Neurosci.* 17 537–549. 10.1038/nrn.2016.70 27334043 PMC5610421

[B25] Younesian S. Yousefi A. M. Momeny M. Ghaffari S. H. Bashash D. (2022). The DNA methylation in neurological diseases. *Cells* 11:3439. 10.3390/cells11213439 36359835 PMC9657829

